# Lessons learned from long-term side effects after zoledronic acid infusion following denosumab treatment: a case report and review of the literature

**DOI:** 10.1186/s13256-022-03695-y

**Published:** 2022-12-16

**Authors:** Ilona Nurmi-Lüthje, Peter Lüthje

**Affiliations:** 1 Department of Public Health, Helsinki University, Mannerheimintie 172, 00300 Helsinki, Finland; 2 Department of Orthopedics and Traumatology, North Kymi Hospital, 45750 Kuusankoski, Finland

**Keywords:** Osteoporosis, Zoledronic acid, Long-term side-effects, Under supervision, Chronic low back pain

## Abstract

**Background:**

Zoledronic acid is an intravenous, highly potent aminobisphosphonate for use in patients with primary or secondary osteoporosis. Zoledronic acid-induced prolonged side-effects are well known and quite common. However, severe side-effects can be a threat to life. We report a case of severe side-effects induced by zoledronic acid infusion, and its positive effect on long-term back pain.

**Case presentation:**

In 2012, a 62-year-old white native Finnish woman was operated on for an estrogen and progesterone receptor-positive breast cancer. After radiotherapy, an aromatase inhibitor (letrozole) was started. Nine months after the operation, the patient suffered a low-energy compression fracture of Th XII. She received denosumab to prevent fragility fractures and to improve bone mineral density. Letrozole was discontinued after 5.5 years, and the last denosumab injection was given after 7 years. Six months later, at the age of 71 years, the patient received a single intravenous zoledronic acid infusion. Suddenly, at 10 hours from the infusion, she complained of severe trismus, muscle twitching, spasms, and tingling, matching hypocalcemia and several other symptoms. Her serum 25-hydroxyvitamin D concentration was high (163 nmol/L), the concentration of serum calcium and calcium-ion was normal (2.32 mmol/L and 1.23 mmol/L, respectively). However, the neutrophil to lymphocyte ratio (NLR) was low (1.6). A complete recovery took 2 months. Zoledronic acid infusion also had a positive effect: for many years, the patient had suffered low back pain and strain, which came to an end after this single infusion.

**Conclusion:**

It is important that the potential patients receive sufficient information about the possibility of side-effects following the administration of intravenous zoledronic acid. To ensure that a zoledronic acid infusion is given as safely as possible, the safety information should include that the patient should not be left without monitoring for a minimum 24 hours after the infusion. Being alone and experiencing serious side-effects may lead to acute cardiac problems. Furthermore, the chronic low back pain and strain that our patient suffered for many years has clearly reduced for 16 months after infusion, so far. We conclude that this is a positive effect of zoledronic acid.

## Introduction

We present a case of long-lasting severe acute phase response (APR) following the administration of intravenous zoledronic acid in a 71-year-old white woman. Zoledronic acid is a nitrogen-containing third-generation bisphosphonate. Bisphosphonates bind to hydroxyapatite in bone, particularly at sites of active bone remodeling, and reduce the activity of bone-resorbing osteoclasts [1]. Intravenous or high-dose oral administration of nitrogen-containing bisphosphonates may cause acute-phase reactions in up to 50% of patients receiving their first dose [2].

Our patient received letrozole medication, an aromatase inhibitor (AI), after the treatment of breast cancer. AIs reduce estrogen levels and are associated with more rapid and greater bone loss compared with normal menopause [3]. Women treated with AIs such as letrozole have higher rates of fractures [3]. According to a recent prospective study, the risk of vertebral fracture during AI therapy was significantly lower for patients receiving denosumab or zolendronic acid therapy compared with oral bisphosphonate therapy [4]. Denosumab and zoledronate can reduce AI-related risk of vertebral fractures already after 24 months of treatment [4].

In all patients initiating AI treatment, fracture risk should be assessed and an antiresorptive therapy should be started for all patients according to the bone mineral density (BMD), with a *T*-score < −2.0 or a *T*-score of < −1.5 standard deviation (SD) with one additional risk factor according to the Fracture Risk Assessment Tool (FRAX algorithm), or with ≥ two risk factors (without BMD) for the duration of AI treatment. Based on current evidence, a denosumab injection biannually for the duration of AI therapy is recommended for the prevention of bone loss in postmenopausal women [3].

Denosumab reduces bone formation and improves bone mineral density (BMD) [5]. Denosumab is a monoclonal antibody against the receptor activator of NF-κB ligand (RANKL) and a potent antiresorptive agent, commonly prescribed for treatment of postmenopausal osteoporosis [5]. In contrast to bisphosphonates, denosumab does not incorporate into bone matrix, and therefore its effects are reversible when therapy is discontinued [6].

In 2015, Papapoulos et al. [7] reported that denosumab treatment for up to 8 years was associated with persistent reductions of bone turnover markers, continued BMD gains, low fracture incidence, and a consistent safety profile. However, in 2016 the first case report of patients who developed new vertebral fractures after discontinuation of denosumab was published [8]. Yet in the previous decade, osteonecrosis of the jaw [9] and atypical femoral fractures [10] emerged as rare potential complications of bisphosphonates and denosumab.

The last European guidance (2019) for the diagnosis and management of osteoporosis in postmenopausal women recommends the use of bisphosphonate therapy after withdrawal of denosumab therapy because of the possible loss of BMD and the risk of vertebral fracture rate [11].

A Swiss 8-year observational study showed that a single infusion of 5 mg zoledronate after a 2–5-year denosumab treatment cycle retained more than half of the gained BMD and was not associated with multiple vertebral fractures, as reported in patients who discontinued denosumab without subsequent bisphosphonate treatment [6].

Unfortunately, the use of a single intravenous zoledronic acid (5 mg/100 mL) treatment has also been associated with mild-to-severe side-effects [12].

We present a case of prolonged severe side-effects and the spontaneous positive effect of zoledronic acid infusion on long-term back pain.

## Case presentation

In November 2012, a white native Finnish woman, aged 62 years at the time, was operated for breast cancer in the left breast (carcinoma ductale, grade 2, size 10 mm). The breast cancer was estrogen and progesterone receptor-positive. Radiotherapy was started 2 months later. Three months after the operation, an aromatase inhibitor (letrozole) was started. At the time there was no national recommendation in Finland for the use for adjuvant bisphosphonate or denosumab treatment during AI therapy, or for measuring BMD in postmenopausal women. The national recommendation for the use of adjuvant denosumab during AI therapy with a 100% special reimbursement started in Finland on 1 March 2017.

In August 2013, the patient stumbled and crashed onto stony ground and got a compression fracture of TH XII. BMD measurements were performed in October 2013 at the lumbar spine (L1–L4), total hip, and femoral neck by DXA (dual-energy X-ray absorptiometry) using a Lunar densitometer (Prodigy, GE Medical Systems). The lowest *T*-scores were at lumbar spine (L1–L2, *T*-scores of –1.9) and in the right femoral neck (*T*-score −1.8).

Because of the aromatase inhibitor (AI) medication and the fracture (compression grade 50%), a subcutaneous denosumab treatment (5 mg every 6 months) was started in October 2013.

Letrozole medication was discontinued after 5.5 years, and the last denosumab injection was given in November 2020. The patient’s additional daily medication was losartan 100 mg, simvastatin 20 mg, levothyroxine 100 mcg, warfarin 6–8 mg depending on the international normalized ratio (INR) level, and daily use of vitamin D supplements 1600 IU in winter and 800 IU in summer months. Permanent warfarin medication was started in 2000 after third distal deep vein thrombosis of the lower limb.

Six months after the last denosumab injection, our then 71-year-old patient received a single intravenous zoledronic acid (Aclasta^R^) infusion (5 mg/100 mL). Due to her permanent use of warfarin medication, she did not receive any nonsteroidal anti-inflammatory drugs (NSAIDs) (for example, indomethacin, acetaminophen, ibuprofen, or diclofenac) before the administration of the infusion. She did not take paracetamol either because its concomitant use with warfarin has been described to increase prothrombin times and bleeding [13]. Her health status was good before this first zoledronic acid infusion.

About 10 hours after the infusion, the patient suddenly suffered an acute phase response (APR), abruptly beginning with severe trismus, muscle twitching, spasms, tingling and numbness in the face and fingers, and ague. This was rapidly followed by influenza-like symptoms such as headache, pain in the pharynx and in the left side of the neck, and continuing within 24 hours with musculoskeletal pain, polyarthralgia, synovitis, abdominal pain, and exertional dyspnea.

A fever appeared quickly and her temperature rose to 39.3 °C and remained over 39.0 °C for 2 days. It began to decline after 3 days, returned to normal after 8 days, and her headache ended after 9 days. Other symptoms continued for 1–3 days but polyarthralgia lasted for nearly 2 months. The diagnosis of APR was undisputed by the patient’s husband who is a general surgeon and a specialist in orthopedics and traumatology.

On the second day, the patient’s husband called an ambulance because of the strong symptoms. Before calling the pre-hospital team, he consulted a general physician who gave the infusion in a private clinic.

The pre-hospital emergency team examined the patient, and because of the normal electrocardiography, oxygen saturation, and blood pressure, the patient remained at home. During the severe APR period she received 2–3 g oral paracetamol daily. There were no other follow-up visits and her physician husband took care of her. The patient’s general fatigue continued for about 2 months.

About 6 months prior to the intravenous zoledronic acid infusion, the following laboratory tests were performed: blood count, sedimentation rate (SR), alkaline phosphatase (ALP), alanine aminotransferase (ALT), parathyroid hormone (PTH), ionized calcium, creatinine, serum 25-hydroxyvitamin D (S-25OHD), thyroid-stimulating hormone (TSH), and free thyroxin (T4-V). All test results were normal (Table 1). Furthermore, certain routine laboratory tests were performed 34 days before the infusion (Table 1). The patient’s international normalized ratio test (INR) was checked every 4–6 weeks. The glomerular filtration rate was 72 mL/min/1.73 m^2^ (reference value over 59 mL/min/1.73 m^2^ for age ≥ 70 years) a day before the infusion. She had no nephropathy.

**Table 1:** Laboratory test results 6 months before and during the first 91 days after the infusion of zoledronic acid

Laboratory test	Reference	15 December 2020	29 April 2021	Day 7	Day 11	Day 54	Day 91
Hemoglobin (g/L)	117–155	130	133	132			136
Leukocyte (E9/L)	3.4–8.2	5.2	6.1	8.0			6.5
Thrombocyte (E9/L)	150–360	245	237	325			288
CRP (mg/L)	< 4			84	41		2
Calcium (mmol/L)	2.15–2.51	2.34	2.30	2.32			
Calcium-ion (mmol/L/ph7.4)	1.16–1.3			1.23			
Creatinine (μmol/L)	50–90	69	73	62			
ALP (U/L)	35–105	44		73			41
Kalium (mmol/L)	3.3–4.9			3.9			4.1
Natrium (mmol/L)	137–145			139			142
SR (mm/h)	< 40	5		47			
S-25OHD (nmol/L)	> 50	139		163			
INR	2–3	2.3	2.2	1.7	2.1	2.1	2.2
PTH (ng/L)	12–47	46		–	45		
TSH (mU/L)	0.3–4.2	1.55		–	10.56	6.30	1.73
T4-V (pmol/L)	11–22	18		–	16	21	20
ALT (U/L)	< 35	23		–			30

*CRP* C-reactive protein, *ALP* alkaline phosphatase, *SR* sedimentation rate, *S-25OHD* serum 25-hydroxyvitamin D, *INR* international normalized ratio, *PTH* parathyroid hormone, *TSH* thyroid-stimulating hormone, *T4-V* free thyroxine, *ALT* alanine aminotransferase

Further laboratory tests were performed at 7, 11, 54, and 91 days after the infusion of zoledronic acid (Table 1). The serum marker of bone resorption test [tartrate-resistant acid phosphatase 5b (TRAP 5b)] [14] was performed at 7 and 12.5 months after the infusion. The results were 3.73 U/L and 3.65 U/L, respectively (range in postmenopausal women: 1.49–4.89 U/L). The patient’s Covid-19 PCR test 9 days after the infusion was negative. All laboratory tests were privately performed at the laboratory of Helsinki University Hospital, Finland.

The last BMD was measured a few days before the zoledronic acid infusion. The BMD of the total lumbar spine (L1–L4) was 14.4% higher than in 2013. The total BMD results in the right total hip was 5.6% higher and in the left total hip 5.4% higher than in 2013.

Written informed consent for publication was obtained from the patient.

## Discussion

APR is common among patients who have never taken bisphosphonates before and are undergoing infusion for the first time [15] Adequate serum 25-hydroxyvitamin D level may be protective for APR [15]. The APR reactions are characterized by fever and muscle aches—a flu-like illness—lasting several days [1]. Acetaminophen, given 1–2 hours before treatment, may reduce the likelihood of these reactions and can also be given to treat the symptoms [1]. According to the Drug Information Database, the APR symptoms usually resolve within 3 days of onset, but resolution can take up to 7–14 days, and some symptoms have persisted for a longer duration [16].

Patients taking denosumab are advised to transition to an oral or intravenous bisphosphonate when the course of therapy is complete, to prevent bone loss and a potential rebound in vertebral fracture risk after discontinuing this drug.

How can we predict the development of APR? Adequate levels of serum 25-hydroxy vitamin D and hydration before zoledronic acid infusion have been reported to lower the incidence of APRs [15, 17]. According to a recent Japanese study, APRs occurred more frequently in osteoporosis patients with a lower neutrophil to lymphocyte ratio (NLR, an indicator for inflammation), higher bone turnover, and younger age [2].

Our patient was 71 years old, had a high serum 25-hydroxyvitamin D level (163 nmol/L) and a normal calcium level (2.32 mmol/L), but low NLR (1.6) (range: low NLR ≤ 3.0 and high > 3.0) [18]. Her NLR between 2014 and 2019 was low (range 0.75–0.83). The level of serum alkaline phosphatase (ALP) to evaluate bone formation and resorption was somewhat higher (73 U/L) a week after the infusion and decreased to 41 U/L after 3 months.

To assess any association between the use of a zoledronic acid infusion (5 mg/100 mL) and thyroid function, we searched eligible studies in MEDLINE up to December 2021. There was only one placebo-controlled prospective study indicating that total serum triiodothyronine (T3), free T3, total thyroxine (T4), and free T4 declined significantly on days 1 and 2 after zoledronic acid infusion, whereas on day 3, levels started to return to the baseline concentrations [19]. Serum TSH remained essentially unchanged [19]. Our patient had a high TSH level (10.56 mU/L) on day 11 after the infusion and the level remained higher than her normal level (1–2 mU/L), even after 53 days (6.30 mU/L). The TSH level was normal at 3 months without changing her daily oral levothyroxine (100 mcg) prescription. According to an Indian study (*n* = 163, 95% postmenopausal women), thyroid functions tests were studied at days 0, 1, 2, 3, 7, and 42 post-zoledronic acid infusion (5 mg) [20]. TSH increased after this infusion and the increase continued until the final 42-day follow-up. The rise in TSH was significantly higher in patients having evidence of thyroid autoimmunity. Our patient was diagnosed with autoimmune thyroid disease about 20 years earlier.

In our patient, the C-reactive protein (CRP) level increased and was still quite high (84 mg/L) at 1 week after the infusion due to the severe APR. Four days later the CRP level was 41 mg/L, and at 3 months the level was normal. According to a Finnish randomized, placebo-controlled, double-blinded study (*n* = 40, mean age 50 years, SD 8.3) on zoledronic acid infusion (5 mg/100 mL) for patients with chronic low back pain and modic changes in lumbar magnetic resonance imaging (MRI), the CRP level was still increased at 1 month after the infusion zoledronic acid [21]. The data also showed that a single intravenous infusion of 5 mg of zoledronic acid was effective in reducing the intensity of low back pain and in reducing the use of NSAIDs within a follow-up time of 1 year [22]. Our patient also experienced that her low back pain and strain she suffered reduced during the first 16 months after infusion. Although the patients in the Finnish study [22] received oral ibuprofen 600 mg or paracetamol 1 g to prevent flu-like symptoms, headache, or fever, and 100,000 IU of vitamin D orally to prevent hypocalcemia, 19/20 (95%) patients versus 7/20 (35%) patients in the placebo group had an APR [22]. In our study, ALP decreased during the follow-up similarly to the Finnish study [21].

Unfortunately, the serum marker test of bone resorption TRAP 5b was not checked before the zoledronic acid infusion. The latter TRAP 5b result over 1 year after the infusion was slightly lower than the former about 7 months after the infusion, and clearly lower than the highest reference value for postmenopausal women (range: 1.49–4.89 U/L). According to our results, zoledronic acid infusion had a positive effect on antiresorptive activity. Serum TRAP 5b is a specific and sensitive marker for monitoring antiresorptive treatment [14].

Some acute symptoms in our patient (muscle twitching, spasms, tingling, and numbness) matched with symptoms of hypocalcemia [23]. However, the serum 25-hydroxyvitamin D concentration in our patient was high (163 nmol/L), and the concentrations of serum calcium and calcium-ion (2.32 mmol/L and 1.23 mmol/L, respectively) and parathyroid hormone (45 ng/L) were normal. Higher baseline serum 25-hydroxyvitamin D levels are found to be protective for APR and hypocalcemia after zoledronic acid infusion [15]. In the recent study among 153 white patients with a postmenopausal osteoporosis who received a zoledronic acid infusion for the first time, none experienced an APR with a serum 25-OHD level over 145 mmol/L [15]. Our patient’s serum 25-hydroxyvitamin D concentration was biannually checked for years. However, her good level of S-25OHD (163 nmol/L) did not protect her from experiencing an APR.

Because APRs are quite common, it is important that the potential patient receive sufficient information about the possibility of an APR following the administration of intravenous zoledronic acid. Our recommendation is that the patient should not be left by him/herself for the first 24 hours after the infusion. Also, the safety information of the zoledronic acid infusion should include this recommendation. Being alone and experiencing a serious APR may lead to acute cardiac problems. For our patient, the APR was sudden and difficult. She was unable to use her mobile phone to call for help if she had been by herself. According to a list of zoledronic acid side-effects, dangerous side-effects such as coma, confusion, convulsions, difficult breathing, hypotension, atrial fibrillation, and cardiac arrhythmia secondary to hypocalcemia are possible [16].

Finally, we suggest studying the possible positive impact of zoledronic acid infusion on back pain among older patients with a low-energy hip or vertebral fracture.

## Conclusions

Mild APR is common with administration of the first zoledronic acid infusion. However, severe APRs, as seen in this case, have been reported. There are no prognostic signs (for example, laboratory methods) to predict, who will develop a severe APR. Therefore, the patient should not be left alone for the first 24 hours after the first zoledronic acid infusion. According to our study, the low neutrophil/lymphocyte ratio before the first infusion may be a prognostic sign for predicting APR.

The possible positive effect of zoledronic acid on long-lasting low back pain among elderly patients requires further studies.

## Data Availability

Data of the patient can be requested from the authors. Do not hesitate to get in touch with the corresponding author if you are interested in these data.
